# Downregulation of intracellular nm23-H1 prevents cisplatin-induced DNA damage in oesophageal cancer cells: possible association with Na^+^, K^+^-ATPase

**DOI:** 10.1054/bjoc.2000.1436

**Published:** 2000-11

**Authors:** N lizuka, K Miyamoto, A Tangoku, H Hayashi, S Hazama, S Yoshino, K Yoshimura, K Hirose, H Yoshida, M Oka

**Affiliations:** Department of Bioregulatory Function, Department of Surgery II, Yamaguchi University School of Medicine, 1-1-1 Minami-kogushi, Ube, Yamaguchi, 755-8505; Molecular Oncology Center, Fukuoka University, 7-45-1 Nanakuma, Jonan-ku, Fukuoka, 814-0180; Biomedical Research Institute, Kureha Chemical Industry, 3-26-2 Hyakunin-cho, Shinjuku-ku, Tokyo, 169; Department of Pharmacy, Yamaguchi University Hospital, 1-1-1 Minami-kogushi, Ube, Yamaguchi, 755-8505, Japan

**Keywords:** nm23, cisplatin, DNA damage, mitochondrial membrane potential, oesophageal squamous cell carcinoma

## Abstract

Previously, we showed that expression of nm23-H1 is associated inversely with sensitivity to cisplatin in human oesophageal squamous cell carcinoma (OSCC). The present study was undertaken to investigate the association of nm23-H1 expression with cisplatin-induced DNA damage in OSCC using antisense nm23-H1 transfectants. YES-2/AS-12, an antisense nm23-H1-transfected OSCC cell line, showed significantly reduced expression of intracellular nm23-H1 protein compared with that in parental YES-2 cells and YES-2/Neo transfectants. Surface expression of nm23-H1 protein was not observed in any of the three cell lines. PCR analysis for DNA damage demonstrated that YES-2/AS-12 cells were more resistant to nuclear and mitochondrial DNA damage by cisplatin than were YES-2/Neo cells. In addition, mitochondrial membrane potentials and DNA fragmentation assays confirmed that YES-2/AS-12 was more resistant than YES-2/Neo to apoptosis induced by cisplatin. In contrast, YES-2/AS-12 was more sensitive to ouabain, a selective inhibitor of Na^+^, K^+^-ATPase, than YES-2 and YES-2/Neo. Pre-treatment with ouabain resulted in no differences in cisplatin sensitivity between the three cell lines examined. Intracellular platinum level in YES-2/AS-12 was significantly lower than that in YES-2 and YES-2/Neo following incubation with cisplatin, whereas ouabain pre-treatment resulted in no differences in intracellular platinum accumulations between the three cell lines. Our data support the conclusion that reduced expression of intracellular nm23-H1 in OSCC cells is associated with cisplatin resistance via the prevention of both nuclear and mitochondrial DNA damage and suggest that it may be related to Na^+^, K^+^-ATPase activity, which is responsible for intracellular cisplatin accumulation. © 2000 Cancer Research Campaign


*British Journal of Cancer* (2000) **83**(9), 1209–1215
© 2000 Cancer Research Campaign
doi: 10.1054/ bjoc.2000.1436, available online at http://www.idealibrary.com on IDEAL®

### Downregulation of intracellular nm23-H1 prevents cisplatin-induced DNA damage in oesophageal cancer cells: possible association with $Na^+$, $K^+$-ATPase

**N Iizuka[1], K Miyamoto[1,3], A Tangoku[2], H Hayashi[2], S Hazama[2], S Yoshino [2], K Yoshimura[2], K Hirose[4], H Yoshida[5] and M Oka[2]**

[1]Department of Bioregulatory Function; [2]Department of Surgery II, Yamaguchi University School of Medicine, 1-1-1 Minami-kogushi, Ube, Yamaguchi 755-8505; [3]Molecular Oncology Center, Fukuoka University, 7-45-1 Nanakuma, Jonan-ku, Fukuoka 814-0180; [4]Biomedical Research Institute, Kureha Chemical Industry, 3-26-2 Hyakunin-cho, Shinjuku-ku, Tokyo 169; [5]Department of Pharmacy, Yamaguchi University Hospital, 1-1-1 Minami-kogushi, Ube, Yamaguchi 755-8505, Japan

**Summary** Previously, we showed that expression of nm23-H1 is associated inversely with sensitivity to cisplatin in human oesophageal squamous cell carcinoma (OSCC). The present study was undertaken to investigate the association of nm23-H1 expression with cisplatin-induced DNA damage in OSCC using antisense nm23-H1 transfectants. YES-2/AS-12, an antisense nm23-H1-transfected OSCC cell line, showed significantly reduced expression of intracellular nm23-H1 protein compared with that in parental YES-2 cells and YES-2/Neo transfectants. Surface expression of nm23-H1 protein was not observed in any of the three cell lines. PCR analysis for DNA damage demonstrated that YES-2/AS-12 cells were more resistant to nuclear and mitochondrial DNA damage by cisplatin than were YES-2/Neo cells. In addition, mitochondrial membrane potentials and DNA fragmentation assays confirmed that YES-2/AS-12 was more resistant than YES-2/Neo to apoptosis induced by cisplatin. In contrast, YES-2/AS-12 was more sensitive to ouabain, a selective inhibitor of $Na^+$, $K^+$-ATPase, than YES-2 and YES-2/Neo. Pre-treatment with ouabain resulted in no differences in cisplatin sensitivity between the three cell lines examined. Intracellular platinum level in YES-2/AS-12 was significantly lower than that in YES-2 and YES-2/Neo following incubation with cisplatin, whereas ouabain pre-treatment resulted in no differences in intracellular platinum accumulations between the three cell lines. Our data support the conclusion that reduced expression of intracellular nm23-H1 in OSCC cells is associated with cisplatin resistance via the prevention of both nuclear and mitochondrial DNA damage and suggest that it may be related to $Na^+$, $K^+$-ATPase activity, which is responsible for intracellular cisplatin accumulation. © 2000 Cancer Research Campaign

**Keywords:** nm23; cisplatin; DNA damage; mitochondrial membrane potential; oesophageal squamous cell carcinoma

Since the initial description of the nm23 gene as an antimetastatic factor whose expression is correlated inversely with tumour metastatic potential in murine melanoma cell lines (Steeg et al, 1988), numerous studies have supported its suppressive effects on tumour metastasis (Leone et al, 1993; Tokunaga et al, 1993; Iizuka et al, 1995; Russell et al, 1998). Different conclusions have been drawn from experiments with a variety of tumours (Higashiyama et al, 1992; Lindmark et al, 1996; Shimada et al, 1998). Thus, the relation of nm23-H1 to tumour metastasis is still controversial, although this discrepancy may be due in part to differences in the specificities of antibodies used. nm 23-H1 encodes nucleoside diphosphate kinase A, which is responsible for the synthesis of most non-ATP nucleoside triphosphates, suggesting that this protein might be involved in a wide variety of biological phenomena in the cell (Otero et al, 1999). Recently, the role of nm23-H1 in sensitivity to anticancer agents has attracted a great deal of attention (Freije et al, 1997). In addition to the antimetastatic property, nm23-H1 has been shown to be associated with sensitivity to cisplatin in breast carcinoma (Ferguson et al, 1996) and ovarian carcinoma (Scambia et al, 1996).

Our recent study of oesophageal squamous cell carcinoma (OSCC) revealed that expression of nm23-H1 protein was associated inversely with prognosis of patients with OSCC after cisplatin-based chemotherapy (Iizuka et al, 1999*a*). Additionally, our in vitro study in OSCC cell lines demonstrated that downregulation of nm23-H1 protein by antisense transfection caused increased resistance to cisplatin but not 5-fluorouracil or etoposide (Iizuka et al, 1999*b*). Ferguson et al (1996) demonstrated increased formation of interstrand DNA cross-links of cisplatin in breast cancer cells that express high levels of nm23-H1. However, the mechanism remains unclear. We hypothesized the specific roles of nm23-H1 in cisplatin-induced cytotoxity on the basis of evidence that there was no association between nm23-H1 status and sensitivity to 5-fluorouracil and etoposide. Because cisplatin-induced cytotoxicity is mainly due to formation of adducts with DNA (Mann et al, 1991), in the present study we used an antisense transfectant of nm23-H1 derived from an OSCC cell line to investigate the association of nm23-H1 with both nuclear and mitochondrial DNA damage induced by cisplatin (Iizuka et al, 1999*b*) and then evaluated the relation of nm23-H1 to apoptosis induced by cisplatin. Additionally, we examined the relation of nm23-H1 expression to $Na^+$, $K^+$-ATPase activity, which contributes to

*Received 10 March 2000*
*Revised 3 July 2000*
*Accepted 5 July 2000*

*Correspondence to:* N Iizuka intracellular accumulation of cisplatin (Andrews et al, 1991; Blok et al, 1999).

### MATERIALS AND METHODS

### Cell lines

YES-2 is a human OSCC cell line established in our laboratory (Oka et al, 1996*a*). YES-2/AS-12 is an antisense-transfected clone that has a 23-fold reduction in the expression level of nm23-H1 protein, and that is approximately 4-fold more resistant to cisplatin than the parental YES-2 cell line and the YES-2/Neo cell line (Iizuka et al, 1999*b*). YES-2 was maintained in Dulbecco's modified Eagle's medium (DMEM) (Nissui, Tokyo, Japan) supplemented with 5% foetal calf serum (FCS), 100 U $ml^{-1}$ penicillin G, and 100 µg $ml^{-1}$ streptomycin. Both YES-2/AS-12 and YES-2/Neo were maintained in the above culture medium containing 200 µg $ml^{-1}$ of G418.

### Assessment of intracellular and surface levels of nm23-H1 protein

For detection of intracellular nm23-H1, $5 \times 10^5$ cells were fixed and then incubated with PBS containing 5% normal mouse serum for 30 min to reduce nonspecific reactions. Subsequently, the cells were treated with 200 µl of permeabilizing solution (Becton Dickinson, San Jose CA, USA) at 37°C for 10 min. After being washed in PBS, they were reacted with 0.5 µg of FITC-conjugated H1-229 (Seikagaku, Tokyo, Japan) at 37°C for 60 min. Finally, measurements of intracellular nm23-H1 levels were made with a Facsort fluorescence-activated cell sorter (Becton Dickinson). FITC-conjugated mouse $IgG^{2a}$ was used as an isotype-matched control for anti-nm23-H1 antibody. Analysis of surface nm23-H1 was also performed using the above method without a permeabilizing solution step.

### PCR analysis of nuclear and mitochondrial DNA damage induced by cisplatin

The cells were seeded into 60-mm tissue culture dishes at 37°C in DMEM with 5% FCS. When the cells were subconfluent, the medium was aspirated and replaced with serum-free medium. Cells were then treated with 25–200 µg $ml^{-1}$ of cisplatin (Nippon Kayaku, Tokyo, Japan). Following incubation for 2 h at 37°C, cells were washed three times with cold PBS, pelleted by centrifugation at 2000 rpm at 4°C and frozen for DNA isolation. Genomic DNA was extracted by DNAzol (Gibco-BRL, Bethesda MD, USA) and the concentration of DNA was determined using a GeneQuant II spectrophotometer (Pharmacia Biotech, Tokyo, Japan). Fifty nanograms of total genomic DNA was amplified by PCR as described by Daoud et al (1995). Namely, a 1.1-kb fragment of mitochondrial DNA was amplified by 18 cycles of PCR with Mito A and Mito B primers (Mito A, 5′-GCGGTTGTTGATGGGTGAGT-3′; Mito B, 5′-CCACAT-CACTCGAGACGTAA-3′) (Yoon et al, 1991), and a 0.536-kb fragment of nuclear *β*-globin gene was amplified by 30 cycles of PCR with the β-globin 1 and β-globin 2 primers (β-globin 1, 5′-GGTTGGCCAATCTACTCCCAGG-3′; β-globin 2, 5′-GCTCACTCAGTGTGGC AAAG-3′) (Saiki et al, 1988). The PCR products were separated by electrophoresis on a 1% agarose gel and stained with ethidium bromide. Quantitative PCR analysis was performed in 50 µl PCR reactions containing 0.5 µl (5 µCi) of α [$^{32}$P]-dCTP as described above. The PCR products were subjected to 5% polyacrylamide gel electrophoresis and autoradiography, and the associated radioactivity was measured with an imaging analyser (BAS-2000) (Fuji Photo Film, Tokyo, Japan).

### Analysis of mitochondrial membrane potential (MMP)

Loss of mitochondrial membrane potential (MMP) was analysed by flow cytometry as previously described (Kim et al, 1997; Fulda et al, 1999). Briefly, $5 \times 10^5$ cells were treated with 0, 2.5 µg $ml^{-1}$, 5 µg $ml^{-1}$, or 10 µg $ml^{-1}$ of cisplatin for 24 h. After cisplatin treatment, cells were resuspended and incubated with 40 nM 3,3′-dihexyloxacarbocyanine iodide (DiOC6(3)) (Molecular Probes Inc, Eugene OR, USA) for 15 min at 37°C. Finally, measurement of MMP was done with a Facsort fluorescence-activated cell sorter (Becton Dickinson) (*see* Figure 4A).

### Analysis of DNA fragmentation

DNA fragmentation assay was performed as previously described (Oka et al, 1996*b*). Briefly, YES-2/Neo and YES-2/AS-12 cells were incubated with 5 µg $ml^{-1}$ of cisplatin for 24 h. After being washed with PBS, the cells were collected and treated with proteinase K (100 µg $ml^{-1}$) and RNase (1 µg $ml^{-1}$). DNA was extracted with DNAzol (Gibco-BRL), and then 3 µg of DNA was separated by electrophresis on 1.5% agarose gels. Finally, the DNA was visualized by staining with ethidium bromide (see Figure 4B).

### Inhibitory effect of ouabain on proliferation

Cells were plated at $5 \times 10^3$ per 100 µl in 96-well plates in DMEM with 5% FCS, and allowed to attach overnight. Three wells were treated with 10–1000 nM of ouabain (Nacalai Tesque, Tokyo, Japan). After 24 h, 3-(4,5-dimethyl-2-thiazol)-2,5-diphenyl-2H tetrazolium bromide (MTT) assay was performed as previously described (Hazama et al, 1999; Iizuka et al, 2000). Cell viability was calculated as the percentage of control cultures that were not exposed to ouabain.

### Effects of ouabain on cisplatin-induced cytotoxicity

Cells were plated at $5 \times 10^3$ per 100 µl in 96-well plates in DMEM with 5% FCS, and allowed to attach overnight. Cells were incubated in the same medium with or without 200 nM ouabain (Nacalai Tesque) for 1 h. The medium was then aspirated and replaced with DMEM containing 5% FCS and 5 µg $ml^{-1}$ of cisplatin or DMEM with 5% FCS. After 48 h, MTT assay was performed. Cell viability was calculated as the percentage of control cultures that were not exposed to both ouabain and cisplatin.

### Cisplatin accumulation

Intracellular platinum (Pt) levels were analysed using a modification of previously described procedure (Ohmori et al, 1994; O'Neill et al, 1999). Briefly, $1 \times 10^6$ cells were incubated in the same medium with or without 200 nM ouabain for 1 h at 37°C.

© 2000 Cancer Research Campaign

After being washed with ice-cold PBS, the cells were exposed to 15 μg $ml^{-1}$ of cisplatin for 2 h under the same conditions. Subsequently, the cells were washed twice with ice-cold PBS, then harvested in 500 μl of PBS and gently sonicated on ice. Protein content was determined using a Bio-Rad Protein Assay Kit (Bio-Rad, Richmond CA, USA). The cell extracts were analysed for platinum using atomic absorption flame emission spectrophotometer (AA-6700F; Shimazu, Tokyo, Japan). The results were expressed as pmol Pt $mg^{-1}$ protein.

### Statistical analysis

The data were analysed by a one-way analysis of variance (ANOVA), and where appropriate, Scheffe's adjustment for multiple comparisons was used. $P < 0.05$ was considered statistically significant.

### RESULTS

### Modulation of intracellular and surface nm23-H1 expression by antisense transfection

When compared to that of controls, the immunoreactivity of intracellular nm23-H1 protein in YES-2, YES-2/Neo, and YES-2/AS-12 cells were 66.1 ± 1.8%, 64.7 ± 1.5%, and 12.8 ± 2.1%, respectively. Thus, YES-2/AS-12, an antisense nm23-H1-transfected OSCC cell line, showed significantly reduced expression of intracellular nm23-H1 protein compared with parental YES-2 and YES-2/Neo ($P < 0.0001$ for both, one-way ANOVA) (Figure 1). These results were consistent with previous data obtained by Western blot analysis (Iizuka et al, 1999*b*). On the other hand, surface expression of nm23-H1 was not detected in the cell lines examined (Figure 1).

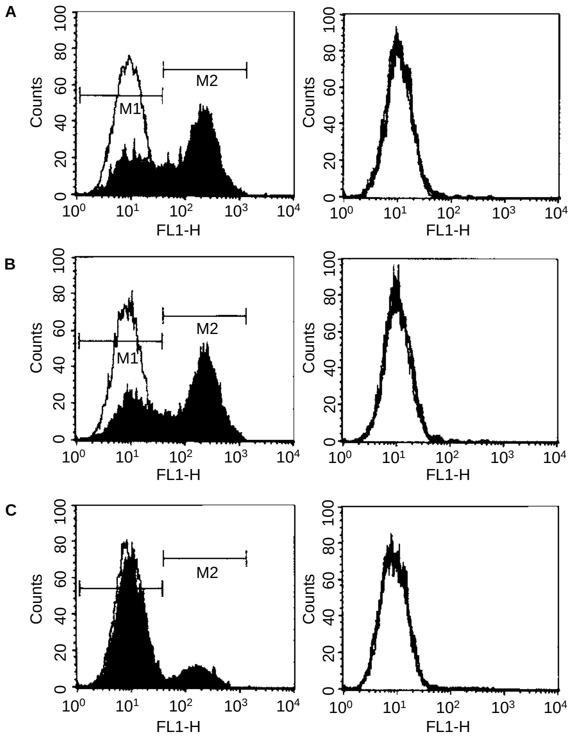

**Figure 1** Intracellular and surface levels of nm23-H1 protein in parental YES-2 cells (**A**), YES-2/Neo cells (**B**), and YES-2/AS-12 cells (**C**). Left and right panels show intracellular and surface expression of nm23-H1 protein, respectively. Shaded histogram (left panel) and bold line (right panel), reactivity with FITC-conjugated H1-229 specific for nm23-H1 protein; thin line (left and right), reactivity with FITC-conjugated mouse $IgG^{2a}$

### Nuclear and mitochondrial DNA damage induced by cisplatin

To evaluate differences in DNA damage caused by cisplatin between YES-2/AS-12 and YES-2/Neo cells, PCR-based assay was performed according to previously described methods (Daoud et al, 1995). This assay is based on the observation that extreme damage to the DNA templates can block the Taq polymerase. Indeed, it was reported that treatment of cells with cisplatin decreased amplification of a specific DNA fragment compared to that of the same DNA fragment in untreated cells (Kalinowski et al, 1992).

In this study, we used primers that amplify a 0.536-kb fragment of the β-globin gene (Saiki et al, 1988) and a 1.1-kb fragment of the mitochondrial DNA (Yoon et al, 1991) to evaluate nuclear and mitochondrial DNA damage induced by cisplatin, respectively. Comparison of ethidium bromide staining showed a significant reduction in amplification of the β-globin gene fragment in YES-2/Neo cells at levels of cisplatin above 50 μg $ml^{-1}$ (Figure 2). On the contrary, amplification of this same fragment of YES-2/AS-12 cells was not inhibited even when the cells were treated with 100 μg $ml^{-1}$ of cisplatin. Amplification of the mitochondrial DNA fragment of YES-2/Neo cells was inhibited by 100 μg $ml^{-1}$ of cisplatin, but amplification of this same fragment of YES-2/AS-12 cells was not affected by 100 μg $ml^{-1}$ of cisplatin. Based on these findings, we used 100 μg $ml^{-1}$ of cisplatin to evaluate quantitatively the difference in DNA damage between YES-2/AS-12 and YES-2/Neo cells. Quantitative PCR showed that after 2-h exposure of YES-2/AS-12 cells to 100 μg $ml^{-1}$ of cisplatin, the level of intact β-globin gene was 93.1% compared to that of untreated cells. This percentage dropped to approximately 17.5% in cisplatin-treated YES-2/Neo cells (Figure 3). Moreover, the level of intact mitochondrial DNA was 95.5% in cisplatin-treated YES-2/AS-12 cells whereas it was 57.9% in cisplatin-treated YES-2/Neo cells. Thus, both nuclear and mitochondrial DNAs of YES-2/AS-12 cells showed increased resistance to cisplatin-induced damage when compared to the results with YES-2/Neo cells. There were no differences in nuclear or mitochondrial DNA damage caused by cisplatin between parental YES-2 and YES-2/Neo cells (data not shown).

### Apoptosis induced by cisplatin in YES-2/Neo and YES-2/AS-12 cells

Flow cytometric analysis using DiOC6(3) showed that 10.7%, 10.7%, 13.4%, and 42.9% of mitochondrial membrane potential (MMP) in YES-2/AS-12 cells was lost after 24-h exposure to 0, 2.5 μg $ml^{-1}$, 5 μg $ml^{-1}$, and 10 μg $ml^{-1}$ of cisplatin, respectively. MMP loss in YES-2/Neo cells was 11.4%, 37.5%, 67.3%, and 86.8%, respectively. (Figure 4A). After 24-h exposure to 5 μg $ml^{-1}$ of cisplatin, DNA fragmentation was detected more strongly in YES-2/Neo than in YES-2/AS-12 cells (Figure 4B).

© 2000 Cancer Research Campaign

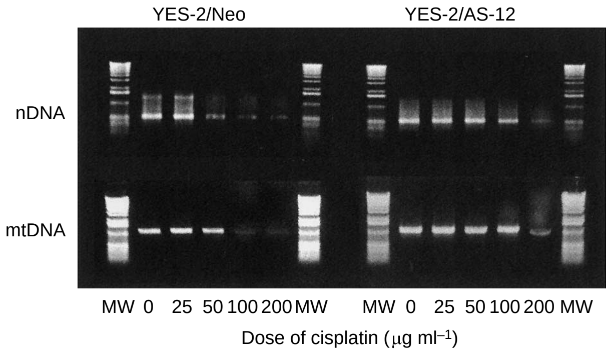

**Figure 2** PCR amplification of nuclear (n) and mitochondrial (mt) DNA damaged by cisplatin in YES-2/Neo cells (left) and YES-2/AS-12 cells (right). The fragment (536 bp) of the genomic β-globin gene and the fragment (1.1 kb) of mitochondrial DNA were amplified by PCR to evaluate nuclear and mitochondrial DNA damage after cisplatin treatment. PCR products were separated electrophoretically on a 1% agarose gel and stained with ethidium bromide. MW = molecular weight marker (1 Kb DNA Ladder, Gibco-BRL, Rockville MD, USA)

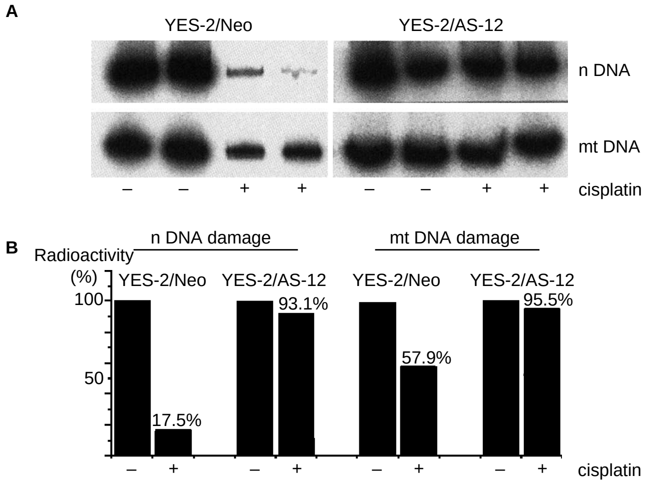

**Figure 3** Quantitative analysis for nuclear (n) and mitochondrial (mt) DNA damage by cisplatin. (**A**) Autoradiograph showing PCR products of genomic β-globin gene (upper panel) and mitochondrial DNA (lower panel) in YES-2/Neo (left) and YES-2/AS-12 (right) cells. Total DNA was extracted from YES-2/Neo and YES-2/AS-12 cells with or without 100 μg ml–1 cisplatin treatment. Subsequently, quantitative PCR analysis was performed as described in 'Materials and Methods'. The PCR products were subjected to 5% polyacrylamide gel electrophoresis and autoradiography, and the associated radioactivity was measured with an imaging analyser (BAS-2000) (Fuji Photo Film, Tokyo, Japan). (**B**) Relative radioactivity is compared to that of non-damaged DNA. Data are mean values of two individual experiments

### Modulation of cisplatin resistance by ouabain

Our present data showed that downregulation of intracellular nm23-H1 expression caused increased resistance to cisplatin-induced DNA damage in OSCC cell lines. Therefore, we examined the relation of nm23-H1 status to the activity of $Na^+$, $K^+$-ATPase, which affects cisplatin influx (Andrews et al, 1991). As shown in Figure 5, after 24-h exposure to ouabain, a selective inhibitor of $Na^+$,$K^+$-ATPase, the proliferation of each cell line was inhibited in a dose-dependent manner. The YES-2/AS-12 cell line was more sensitive to ouabain than were the YES-2 and YES-2/Neo cell lines at doses of 100–1000 nM ($P<0.001$ for both, one-way ANOVA). As shown in Table 1, after 48-h exposure to cisplatin without ouabain pretreatment, cell viability of YES-2/AS-12 was 47.9 ± 2.2% when compared to that of untreated

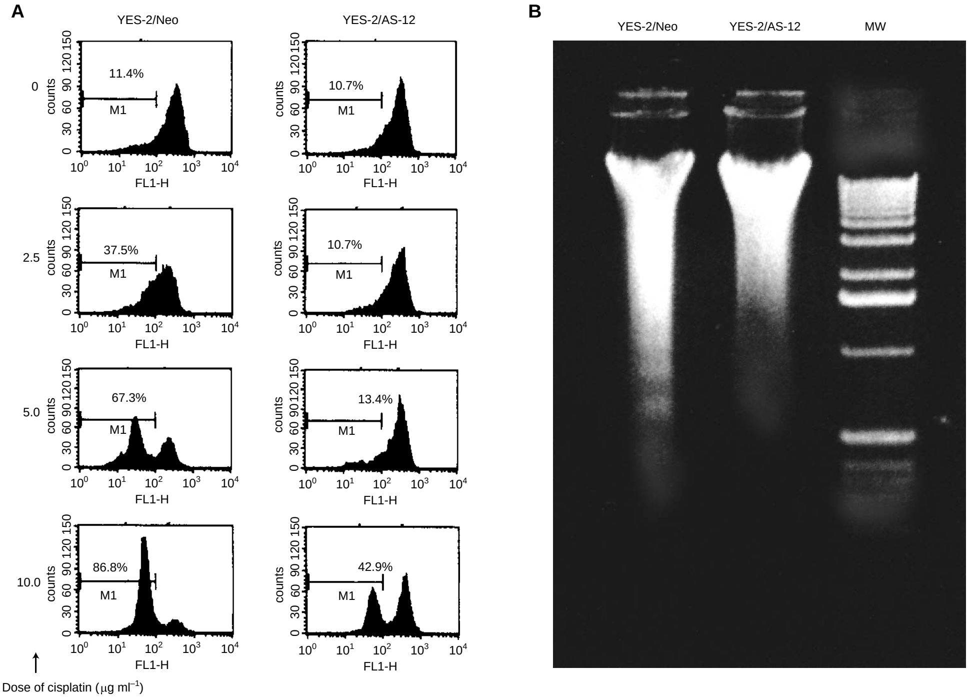

**Figure 4** Apoptosis induced by cisplatin in YES-2/Neo and YES-2/AS-12 cells. (**A**) Loss of mitochondrial membrane potential (MMP) in YES-2/Neo (left) and YES-2/AS-12 (right) cells. Cells were treated with 0, 2.5, 5 or 10 μg ml–1 of cisplatin for 24 h. They were resuspended and incubated with 40 nM DiOC6(3) for 15 min at 37°C. Finally, loss of MMP was measured with a Facsort fluorescence-activated cell sorter (Becton Dickinson). (**B**) DNA fragmentation induced by 5 μg ml–1 of cisplatin for 24 h. MW = molecular weight marker (1 Kb DNA Ladder, Gibco-BRL).

 © 2000 Cancer Research Campaign

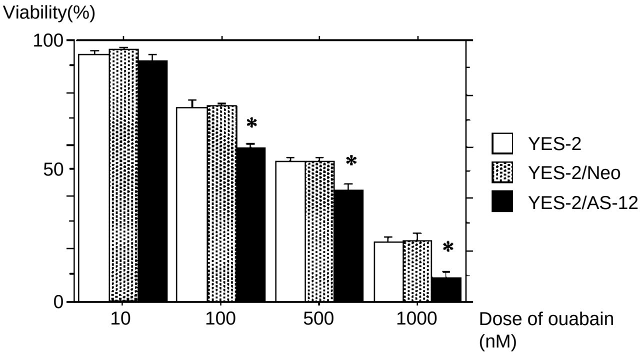

**Figure 5** Inhibitory effects of ouabain on proliferation of OSCC cell lines. Cells were treated with 10–1000 nM of ouabain. After 24 h, MTT assay was performed as described in 'Materials and Methods'. Cell viability was calculated as the percentage of control cultures which were not exposed to ouabain. Data are mean values ± SE of three individual experiments (*P < 0.001 vs YES-2 or YES-2/Neo)

cells. Cell viabilities of YES-2 and YES-2/Neo were 14.8 ± 3.1% and 16.1 ± 2.4%, respectively. Thus, YES-2/AS-12 showed significantly increased resistance to cisplatin as compared to YES-2 and YES-2/Neo ($P$ = 0.0009 and $P$ = 0.0006, respectively, one-way ANOVA). This result was consistent with our previous data (Iizuka et al, 1999*b*). In contrast, after 48-h exposure to cisplatin with ouabain pre-treatment, cell viabilities of YES-2, YES-2/Neo, and YES-2/AS-12 were 54.1 ± 1.7%, 54.3 ± 1.6%, and 57.4 ± 2.4%, respectively. Thus, there was no significant difference in cisplatin sensitivity between the three cell lines when they were pretreated with 200 nM ouabain (Table 1). After 2-h exposure to 15 μg ml$^{-1}$ of cisplatin, the amount of platinum accumulated in YES-2, YES-2/Neo, and YES-2/AS-12 were 184.5± 24.8, 189.6± 21.0, and 95.0 ± 13.3 pmol mg$^{-1}$ protein, respectively. Thus, intracellular platinum level in YES-2/AS-12 was significantly lower than that in YES-2 and YES-2/Neo ($P$ = 0.029 and $P$ = 0.019, respectively), whereas ouabain pre-treatment resulted in no differences in intracellular platinum levels between the three cell lines (Table 2).

### DISCUSSION

Cisplatin is a widely used anticancer agent that acts by forming adducts with DNA and initiating the response to cellular injury (Andrews et al, 1991; Mann et al, 1991). For treatment of oesophageal squamous cell carcinoma (OSCC), however, cisplatin-based chemotherapy has not led to significant improvement in overall survival of OSCC patients (Kok et al, 1996; Bosset et al, 1997). Therefore, it is necessary to elucidate the mechanism of cisplatin resistance or sensitivity of OSCC. In addition to numerous factors responsible for cisplatin resistance, our recent study using the MTT assay revealed that downregulation of nm23-H1 protein increases the resistance of OSCC cell lines to cisplatin but not to 5-fluorouracil or etoposide (Iizuka et al, 1999*b*). Our present data demonstrate that downregulation of intracellular nm23-H1 increases cellular resistance to cisplatin-induced DNA damage in OSCC cells. Ferguson et al (1996) proposed that overexpression of nm23-H1 increases formation of interstrand DNA cross-links of cisplatin in human breast cancer cells. Therefore, it is possible that the increased resistance to cisplatin-induced DNA damage in YES-2/AS-12 cells is due in part to altered formation of interstrand DNA cross-links of cisplatin. Our data also demonstrate that downregulation of intracellular nm23-H1 increases resistance to cisplatin-induced mitochondrial DNA damage in OSCC cells. The relation of nm23-H1 to mitochondrial DNA damage indicates the significance of nm23-H1 in cisplatin-induced cytotoxicity, because mitochondrial injury has been shown to be a central event in the early stages of cell death (Olivero et al, 1995) and it has been shown that there is decreased removal of cisplatin-DNA adducts in damaged mitochondrial DNA compared to damaged nuclear DNA (Olivero et al, 1997). In addition, alterations in mitochondrial functions, such as permeability transitions, play a central role in the apoptotic process (Kroemer et al, 1998; Eguchi et al, 1999). We demonstrate that downregulation of intracellular nm23-H1 decreases cisplatin-induced loss of mitochondrial membrane potential and DNA fragmentation. This result might be due to the difference in mitochondrial DNA damage in the OSCC cell lines.

We confirmed that downregulation of nm23-H1 did not alter the expression of GST-π and metalothionein mRNAs (data not shown). Since these genes or proteins have been reported to be associated with cellular detoxification in cisplatin-induced cytotoxicity and have been demonstrated to be elevated in cisplatin resistance cell lines (Timmer-Bosscha et al, 1993; Hishikawa et al, 1997), this result suggested the contribution of nm23-H1 to another mechanism in cisplatin resistance other than cellular detoxification. Cisplatin uptake is also thought to be associated with the activity of $Na^+$ $K^+$-ATPase (Andrews et al, 1991; Mann et al, 1991; Blok et al, 1999). Increased sensitivity to ouabain, a selective inhibitor of $Na^+$,$K^+$-ATPase, is observed in cisplatin-resistant cancer cells (Ohmori et al, 1994), suggesting that lower $Na^+$, $K^+$-ATPase activity is essential for cisplatin resistance. On the contrary, cisplatin-resistant cancer cells were reported to be cross-resistant to ouabain (Andrews et al, 1991). This discrepancy might be due in part to difference in the cancer cell type used. Consistent

**Table 1** Modulation of cisplatin sensitivity by ouabain pre-treatment in oesophageal cancer cells

| | Cell viability (%) | | |
|---|---|---|---|
| | Ouabain ( + ) | | Ouabain (−) |
| Treatment | Cisplatin (−) | Cisplatin (+) | Cisplatin (+) |
| YES-2 | 91.9 ± 2.0* | 54.1 ± 1.7** | 14.8 ± 3.1*** |
| YES-2/Neo | 91.4 ± 3.2* | 54.3 ± 1.6** | 16.1 ± 2.4*** |
| YES-2/AS-12 | 86.7 ± 2.3* | 57.4 ± 2.4** | 47.9 ± 2.2*** |

Data are mean values ± SE of three individual experiments.
*, **Not significant. ***$P$ = 0.0009 (YES-2/AS-12 *versus* YES-2);
***$P$ = 0.0006 (YES-2/AS-12 *versus* YES-2/Neo).

**Table 2** Cellular accumulation of cisplatin and inhibitory effect of ouabain

| | Cisplatin accumulation (pmol mg$^{-1}$ protein) | |
|---|---|---|
| Pre-treatment of Ouabain | (−) | (+) |
| YES-2 | 184.5 ± 24.8* | 99.5 ± 7.3** |
| YES-2/Neo | 189.6 ± 21.0* | 105.1 ± 10.2** |
| YES-2/AS-12 | 95.0 ± 13.3* | 80.4 ± 9.5** |

Data are mean values ± SE of four individual experiments.
*$P$=0.029 (YES-2/As-12 *versus* YES-2); *$P$ = 0.019 (YES-2/AS-12 *versus* YES-2/Neo). **Not significant.

© 2000 Cancer Research Campaign with the results by Ohmori et al (1994), our results showed that YES-2/AS-12 cells were more sensitive to ouabain than were YES-2 and YES-2/Neo cells. Intracellular platinum level in YES-2/AS-12 was significantly lower than that in YES-2 and YES-2/Neo following incubation with cisplatin. The amount of platinum accumulated in YES-2/AS-12 was about 50% of that in YES-2 or YES-2/Neo. This result is also supported by the report that approximately half of the cisplatin accumulation in the cell is due to $Na^+$,$K^+$-ATPase (Ohmori et al, 1994). Additionally, inhibition of $Na^+$,$K^+$-ATPase by ouabain pre-treatment resulted in no differences in intracellular platinum accumulations between the three cell lines. These findings support the possibility that activity of $Na^+$,$K^+$-ATPase is lower in YES-2/AS-12 cells than in YES-2 and YES-2/Neo cells. Taken together, close relation of intracellular nm23-H1 to $Na^+$,$K^+$-ATPase activity may confer cisplatin resistance by modulating intracellular accumulation of cisplatin. The relation between nm23-H1 expression and $Na^+$, $K^+$-ATPase activity is not well understood. Nm23/nucleoside diphosphate kinases are ubiquitous enzymes which produce nucleoside triphosphates other than ATP. Under physiological conditions, the reaction occurs at the expense of ATP as a phosphate donor (Ishikawa et al, 1992). Thus, the possible interaction between the two enzymes may be explained in part by the fact that nm23/nucleoside diphosphate kinase and $Na^+$,$K^+$-ATPase function as ATP-scavenging enzymes. It was also shown that $Na^+$ and $K^+$ regulate phosphorylation of nm23 in human airway epithelium (Marshall et al, 1999). Thus, the two enzymes might be associated closely by intracellular cation levels. Further studies are necessary to elucidate possible interaction between these two enzymes at the mRNA and protein levels.

Urano et al (1993) demonstrated the presence of nm23-H1 on the surface of some cancer cells, indicating a possible extracellular role of this protein. In addition, Zaborina et al (1999) showed secretion of nucleoside diphosphate kinase from macrophages stimulated by *Mycobacterium bovis BCG*. In the present study, however, expression of nm23-H1 was not observed on the surface of the three OSCC examined. Additionally, nm23-H1 was not detected on the surfaces of the other five OSCC cell lines (data not shown). Thus, with respect to OSCC, surface nm23-H1 does not exist or function biologically.

Finally, our data support the conclusion that reduced expression of intracellular nm23-H1 in OSCC cells is associated with cisplatin resistance via the prevention of both nuclear and mitochondrial DNA damage. This is due in part to decreased activity of $Na^+$,$K^+$-ATPase, which is responsible for intracellular cisplatin accumulation. These findings suggest the participation of nm23-H1 in antitumour activity of cisplatin as a new biological role of this protein in addition to the antimetastatic property. Thus, modulation of intracellular nm23-H1 in tumour cells may be a novel strategy to maximize the effect of cisplatin-based chemotherapy for OSCC.

### ACKNOWLEDGEMENTS

This work was supported by Grants from Tsumura and Co and from the Ministry of Education, Science, Sports and Culture of Japan.

### REFERENCES

Andrews PA, Mann SC, Huynh HH and Albright KD (1991) Role of the Na+, K(+)-adenosine triphosphatase in the accumulation of cis-diamminedichloroplatinum(II) in human ovarian carcinoma cells. *Cancer Res* **51**: 3677–3681

Blok LJ, Chang GT, Steenbeek-Slotboom M, van Weerden WM, Swarts HG, De Pont JJ, van Steenbrugge GJ and Brinkmann AO (1999) Regulation of expression of Na+, K+-ATPase in androgen-dependent and androgen-independent prostate cancer. *Br J Cancer* **81**: 28–36

Bosset JF, Gignoux M, Triboulet JP, Tiret E, Mantion G, Elias D, Lozach P, Ollier JC, Pavy JJ, Mercier M and Sahmoud T (1997) Chemoradiotherapy followed by surgery compared with surgery alone in squamous-cell cancer of the esophagus. *N Engl J Med* **337**: 161–167

Daoud SS, Clements MK and Small CL (1995) Polymerase chain reaction analysis of cisplatin-induced mitochondrial DNA damage in human ovarian carcinoma cells. *Anti-Cancer Drugs* **6**: 405–412

Eguchi Y, Srinivasan A, Tomaselli KJ, Shimizu S and Tsujimoto Y (1999) ATP-dependent steps in apoptotic signal transduction. *Cancer Res* **59**: 2174–2181

Ferguson AW, Flatow U, MacDonald NJ, Larminat F, Bohr VA and Steeg PS (1996) Increased sensitivity to cisplatin by nm23-transfected tumor cell lines. *Cancer Res* **56**: 2931–2935

Freije JM, Lawrence JA, Hollingshead MG, De la Rosa A, Narayanan V, Grever M, Sausville EA, Paull K and Steeg PS (1997) Identification of compounds with preferential inhibitory activity against low-Nm23-expressing human breast carcinoma and melanoma cell lines. *Nature Med* **4**: 395–401

Fulda S, Lutz W, Schwab M and Debatin KM (1999) MycN sensitizes neuroblastoma cells for drug-induced apoptosis. *Oncogene* **18**: 1479–1486

Hazama S, Noma T, Wang F, Iizuka N, Ogura Y, Yoshimura K, Inoguchi E, Hakozaki M, Hirose K, Suzuki T and Oka M (1999) Tumour cells engineered to secrete interleukin-15 augment anti-tumour immune responses in vivo. *Br J Cancer* **80**: 1420–1426

Higashiyama M, Doi O, Yokouchi H, Kodama K, Nakamori S, Tateishi R and Kimura N (1992) Immunohistochemical analysis of nm23 gene product/NDP kinase expression in pulmonary adenocarcinoma: Lack of prognostic value. *Br J Cancer* **66**: 533–536

Hishikawa Y, Abe S, Kinugasa S, Yoshimura H, Monden N, Igarashi M, Tachibana M and Nagasue N (1997) Overexpression of metallothionein correlates with chemoresistance to cisplatin and prognosis in esophageal cancer. *Oncology* **54**: 342–347

Iizuka N, Oka M, Noma T, Nakazawa A, Hirose K and Suzuki T (1995) NM23-H1 and NM23-H2 messenger RNA abundance in human hepatocellular carcinoma. *Cancer Res* **55**: 652–657

Iizuka N, Tangoku A, Hayashi H, Yosino S, Abe T, Morioka T and Oka M (1999*a*) The association between nm23-H1 expression and survival in patients with esophageal squamous cell carcinoma. *Cancer Lett* **138**: 139–144

Iizuka N, Hirose K, Noma T, Hazama S, Tangoku A, Hayashi H, Abe T, Yamamoto K and Oka M (1999*b*) The nm23-H1 gene as a predictor of sensitivity to chemotherapeutic agents in oesophageal squamous cell carcinoma. *Br J Cancer* **81**: 469–475

Iizuka N, Miyamoto K, Okita K, Tangoku A, Hayashi H, Yosino S, Abe T, Morioka T, Hazama S and Oka M (2000) Inhibitory effect of Coptidis Rhizoma and berberine on the proliferation of human esophageal cancer cell lines. *Cancer Lett* **148**: 19–25

Ishikawa N, Shimada N, Munakata Y, Watanabe K and Kimura N (1992) Isolation and characterization of a gene encoding rat nucleoside diphosphate kinase. *J Biol Chem* **267**: 14366–14372

Kalinowski DP, Illenye S and Van Houten B (1992) Analysis of DNA damage and repair in murine leukemia L1210 cells using a quantitative polymerase chain reaction assay. *Nucleic Acids Res* **20**: 3485–3494

Kim CN, Wang X, Huang Y, Ibrado AM, Liu L, Fang G and Bhalla K (1997) Overexpression of Bcl-X(L) inhibits Ara-C-induced mitochondrial loss of cytochrome c and other perturbations that activate the molecular cascade of apoptosis. *Cancer Res* **57**: 3115–3120

Kok TC, Van der Gaast A, Dees J, Eykenboom WM, Van Overhagen H, Stoter G, Tilanus HW and Splinter TA (1996) Cisplatin and etoposide in oesophageal cancer: a phase II study. *Br J Cancer* **74**: 980–984

Kroemer G, Dallaporta B and Resche-Rigon M (1998) The mitochondrial death/life regulator in apoptosis and necrosis. *Annu Rev Physiol* **60**: 619–642

Leone A, Flatow U, VanHoutte K and Steeg PS (1993) Transfection of human nm23-H1 into the human MDA-MB-435 breast carcinoma cell line: effects on tumor metastatic potential, colonization and enzymatic activity. *Oncogene* **8**: 2325–2333

Lindmark G (1996) NM-23 H1 immunohistochemistry is not useful as predictor of metastatic potential of colorectal cancer. *Br J Cancer* **74**: 1413–1418

Mann SC, Andrews PA and Howell SB (1991) Modulation of cis-diamminedichloroplatinum (II) accumulation and sensitivity by forskolin and 3-isobutyl-1-methylxanthine in sensitive and resistant human ovarian carcinoma cells. *Int J Cancer* **48**: 866–872

© 2000 Cancer Research Campaign

Marshall LJ, Muimo R, Riemen CE and Mehta A (1999) Na+ and K+ regulate the phosphorylation state of nucleoside diphosphate kinase in human airway epithelium. *Am J Physiol* **276**: C109–119

Ohmori T, Nishio K, Ohta S, Kubota N, Adachi M, Komiya K and Saijo N (1994) Ouabain-resistant non-small-cell lung-cancer cell line shows collateral sensitivity to cis-diamminedichloroplatinum(II) (CDDP). *Int J Cancer* **57**: 111–116

Oka M, Iizuka N, Yamamoto K, Gondo T, Abe T, Hazama S, Akitomi Y, Koshihara Y, Ohsugi Y, Ooba Y, Ishihara T and Suzuki T (1996*a*) The influence of interleukin-6 on the growth of human esophageal cancer cell lines. *J Interferon Cytokine Res* **16**: 1001–1006

Oka M, Hirazawa K, Yamamoto K, Iizuka N, Hazama S, Suzuki T and Kobayashi N (1996b) Induction of Fas-mediated apoptosis on circulating lymphocytes by surgical stress. *Ann Surg* **223**: 434–440

Olivero OA, Semino C, Kassim A, Lopez-Larraza DM and Poirier MC (1995) Preferential binding of cisplatin to mitochondrial DNA of Chinese hamster ovary cells. *Mutation Res* **346**: 221–230

Olivero OA, Chang PK, Lopez-Larraza DM, Semino-Mora MC and Poirier MC (1997) Preferential formation and decreased removal of cisplatin-DNA adducts in Chinese hamster ovary cell mitochondrial DNA as compared to nuclear DNA. *Mutation Res* **391**: 79–86

O'Neill CF, Koberle B, Masters JRW and Kelland LR (1999) Gene-specific repair of Pt/DNA lesions and induction of apoptosis by the oral platinum drug JM216 in three human ovarian carcinoma cell lines sensitive and resitant to cisplatin. *Br J Cancer* **81**: 1294–1303

Otero AS, Doyle MB, Hartsough MT and Steeg PS (1999) Wild-type NM23-H1, but not its S120 mutants, suppresses desensitization of muscarinic potassium current. *Biochim Biophys Acta* **1449**: 157–168

Russell RL, Pedersen AN, Kantor J, Geisinger K, Long R, Zbieranski N, Townsend A, Shelton B, Brunner N and Kute TE (1998) Relationship of nm23 to proteolytic factors, proliferation and motility in breast cancer tissues and cell lines. *Br J Cancer* **78**: 710–717

Saiki RK, Gelfand DH, Stoffel S, Scharf SJ, Higuchi R, Horn GT, Mullis KB and Erlich HA (1988) Primer-directed enzymatic amplification of DNA with a thermostable DNA polymerase. *Science* **239**: 487–491

Scambia G, Ferrandina G, Marone M, Benedetti PP, Giannitelli C, Piantelli M, Leone A and Mancuso S (1996) Nm23 in ovarian cancer: correlation with clinical outcome and other clinicopathologic and biochemical prognostic parameters. *J Clin Oncol* **14**: 334–342

Shimada M, Taguchi K, Hasegawa H, Gion T, Shirabe K, Tsuneyoshi M and Sugimachi K (1998) Nm23-H1 expression in intrahepatic or extrahepatic metastases of hepatocellular carcinoma. *Liver* **118**: 337–342

Stahl JA, Leone A, Rosengard AM, Potter L, King CR and Steeg PS (1991) Identification of a second human nm23 gene, nm23-H2. *Cancer Res* **51**: 445–449

Steeg PS, Bevilacqua G, Kopper L, Thorgeirsson UP, Talmadge JB, Liotta LA and Sobel M (1988) Evidence for a novel gene associated with low tumor metastatic potential. *J Natl Cancer Inst* **80**: 200–204

Timmer-Bosscha H, Timmer A, Meijer C, de Vries EG, de Jong B, Oosterhuis JW and Mulder NH (1993) cis-diamminedichloroplatinum (ii) resistance in vitro and in vivo in human embryonal carcinoma cells. *Cancer Res* **53**: 5707–5713

Tokunaga Y, Urano T, Furukawa K, Kondo K, Kanematsu T and Shiku H (1993) Reduced expression of nm23-H1, but not of nm23-H2 is concordant with the frequency of lymph node metastasis of human breast cancer. *Int J Cancer* **55**: 66–71

Urano T, Furukawa K and Shiku H (1993) Expression of nm23/NDP kinase proteins on the cell surface. *Oncogene* **8**: 1371–1376

Willems R, Van Bockstaele DR, Lardon F, Lenjou M, Nijs G, Snoeck HW, Berneman ZN and Slegers H (1998) Decrease in nucleoside diphosphate kinase (NDPK/nm23) expression during hematopoietic maturation. *J Biol Chem* **273**: 13663–13668

Yoon KL, Modica-Napolitano JS, Ernst SG and Aprille JR (1991) Denaturing gradient gel method for mapping single base changes in human mitochondrial DNA. *Anal Biochem* **196**: 427–432

Zaborina O, Li X, Cheng G, Kapatral V and Chakrabarty AM (1999) Secretion of ATP-utilizing enzymes, nucleoside diphosphate kinase and ATPase, by *Mycobacterium bovis* BCG: sequestration of ATP from macrophage P2Z receptors? *Mol Microbiol* **31**: 1333–1343

*© 2000 Cancer Research Campaign*
